# The impact of non-structural carbohydrates (NSC) concentration on yield in *Prunus dulcis, Pistacia vera*, and *Juglans regia*

**DOI:** 10.1038/s41598-022-08289-8

**Published:** 2022-03-14

**Authors:** Maciej A. Zwieniecki, Anna M. Davidson, Jessica Orozco, Katelyn B. Cooper, Paula Guzman-Delgado

**Affiliations:** 1 Department of Plant Sciences, University of California Davis, Davis, CA 95616 USA; 2 Department of Natural Resources and the Environment, Cornell University, Ithaca, NY 14853 USA

**Keywords:** Plant sciences, Environmental sciences

## Abstract

Successful yield in orchards is the culmination of a series of events that start with plants entering dormancy with adequate energy reserves (non-structural carbohydrates; NSC). These NSC are responsible for the maintenance of activities during dormancy and extending onto the period of activeness. Using multi-year yield information and monthly NSC content in twigs, we show that high levels of carbohydrate in *Prunus dulcis*, *Pistachio vera*, and *Juglans regia* during the winter months are indeed associated with high yield, while high levels of the NSC in late summer often correlate with low yield. An evaluation of monthly NSC level importance on yield revealed that for *P. dulcis* high levels in February were a good predictor of yield and that low levels throughout summer were associated with high yield. In *P. vera*, high levels of NSC in December were best predictors of yield. *J. regia* exhibited peculiar patterns; while high pre-budbreak reserves were associated with high yields they only played a minor role in explaining crop, the most important months for predicting yields were June and July. Results suggest that NSC levels can serve as good predictors of orchard yield potential and should be monitored to inform orchard management.

## Introduction

*“path to literary perfection—small alliteration”* JO

Perennials are characterized by their persistence across seasonal cycles, including recurring periods of dormancy and activity, that may be intermittently punctuated by biotic and abiotic disturbances. As such, they must accommodate both short and long-term fluctuations in energy supply and demand^1–4^. The nonstructural carbohydrates (NSC) reserve pool in trees, mostly consisting of soluble sugars and starch, constitutes the primary resource-supply for energetic disparities^3,4^. Short-term variability typically results from the day/night cycle wherein metabolic processes continuously draw energy from NSC reserves which are all the while replenished by the daily photosynthetic activity^5^. Long-term variability can result from either periods of drought, forcing plants to keep stomata closed (days–weeks), or from seasonal shifts like dormancy when photosynthetic activity is relatively absent (summer–winter). Thus, the survival of perennial species depends on their ability to accumulate adequate NSC reserves to meet resource demands during any period in which photosynthetic output is absent or lacking.

Furthermore, not only are sufficient NSC reserves critical for supplementing energy deficiencies but a growing body of evidence suggests that whole-tree NSC reserve levels/status play an important role in sustaining and synchronizing certain phenological progressions. NSC reserves, specifically those present when approaching the end of the season/dormancy, can affect all aspects of tree physiology including effective bloom, spring growth, and ultimately yield. The level of NSC in twigs, in particular, seems to be the most variable yet the most indicative of whole tree storage status^3^ and is of major importance in terms of energy supply for flower development and during the initial phases of vegetative bud growth in species like *Prunus dulcis*, *Pistacia vera*, and *Juglans regia*^1,6^. Additionally, there is increasing evidence suggesting that conversions between NSC forms, soluble sugars and starches, may potentially serve as a ‘dormancy clock’^6^. Hence, the timing and synchrony of bloom is strongly impacted by disturbances especially to the NSC accumulation and dispersion that flank dormancy^7,8^.

Surprisingly, despite the importance of NSC activity in providing protection against adverse weather conditions through dormancy and in influencing the timing and synchrony of bloom, little attention has been given to how trees physiologically prepare for this quintessential period of quiescence^6,7,9^. The amount of reserves needed to maintain dormancy and a healthy growth resumption (bloom/leafing) can be variable and not easily predicted. The build-up of pre-dormancy reserves may be subject to changing abiotic and biotic conditions, growth, and reproductive activity during the active season^3^ especially in alternate bearing species like *P. vera*. Additionally, not only is the NSC reservoir contingent upon the active season but the length and conditions of the dormant period itself can also vary from year-to-year, further affecting the amount of reserves readily available to sustain phenological transitions. The unpredictable nature of the local climate combined with the selection for yield maximization most likely enforce the need to store more NSC reserves in domesticated plants than what is required for the average dormancy period in most undomesticated perennials^10,11^. While the accumulation of reserves is often seen as a byproduct of an excess of carbohydrates, mounting evidence suggests that it may actually be a sink that actively competes with growth and reproduction rather than merely a passive process^12,13^. To their undomesticated counterparts, this ‘excess’ might provide a competitive advantage in which long-term survival is promoted over current vegetative growth and reproductive capacity (yield). However, in domesticated fruit and nut species this may instead shift to promoting short-term gains in reproductive capacity in lieu of long-term NSC reserve formation. This, in turn, potentially makes selected varieties potentially more susceptible to the negative impacts of unexpected changes in dormancy conditions and reduces their resilience to additional stresses. Finally, as a healthy and synchronous bloom is a prerequisite for pollination and fruit set, any changes to NSC content and its forms as affected by weather, biological stress or management can result in significant yield variation. Furthermore, since NSC levels and their form can affect a range of physiological activities, it is important to ask if and when NSC content has the greatest impact on tree productivity and if it is always better to assure high NSC content to generate high yields. Therefore, to answer these questions, we used multi-year observations of NSC content in twigs of *P. dulcis*, *P. vera*, and *J. regia* and combined them with reported yields for over 300 orchards located across the Central Valley, CA, USA.

## Materials and methods

**Figure 1:**
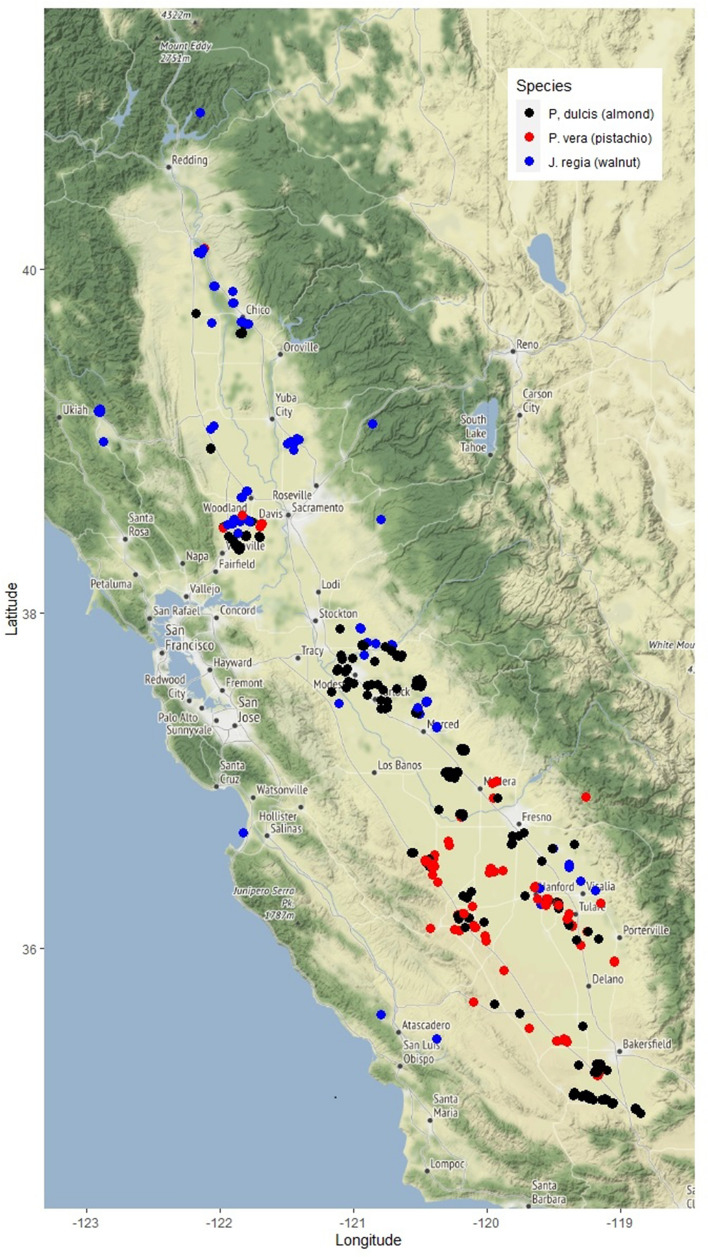
Geographical location of orchards used in the analysis. Figure was generated using R version 3.6.3 with packages ggplot2 and ggmap (Google Maps).

Using a Citizen Science approach, growers across the entire Central Valley of California sent samples of current-season twigs of *Prunus dulcis* (Mill. D.A Webb), *Pistacia vera* L. and *Juglans regia* L. (Fig. 1). The study complies with local and national guidelines. The carbohydrate data set used in this study spans from September 2016 to August 2019 with yield data for the 2017–2019 period. Out of over 590 orchards participating in the NSC study, we selected the orchards from which growers shared yield information for at least one year during the 2017–2019 period. This resulted in 132 *P. dulcis*, 122 *P. vera*, and 84 *J. regia* orchards used in the presented analysis. We encouraged growers to collect samples once a month, however frequency and participation level varied over time and therefore the data sets varied from month to month.

Specific details of sample collection and handling were described previously^1^. Briefly, a unified protocol for sample collection required that one current season twig from three trees per orchard be cut at the base where the current season’s wood met last year’s wood. The bark from the lower 10 cm of the twig was removed using a razor blade. Both the bark and the wood of the three twigs were put in a paper envelope and mailed to the laboratory for NSC analysis. Buds were excluded from the samples. The integrity of the NSC content over shipping time was tested to assure the quality of the results^1^. Upon arrival, samples were put in the dryer for 48 h at 75 °C. The bark and wood were chopped into small < 1 mm pieces separately and ~ 100 mg of each was ground into a fine powder (~ 1 µm) using a ball grinder (MiniBeadbeater-96, Glen Mills Inc., NJ). To analyze for soluble sugar and starch content, we used the previously described protocol^14^ with modifications to use smaller sample sizes^15^. Specifically, 25 mg of powder per sample was placed in 1.5 mL tubes. Tubes were then treated with 1 mL of sodium acetate buffer (0.2 M, pH 5.5), vortexed, and incubated in a 70 °C water bath for 15 min and centrifuged (10 min at 21,000*g*). 50 µL of supernatant was extracted and diluted in ultra-pure (UP) water (1:20, v:v) and vortexed. Soluble sugar content was quantified from diluted supernatant tubes using an anthrone/sulfuric acid colorizing reagent (0.1% (m:v) in 98% sulfuric acid) and reading absorbance at 620 nm in a spectrophotometer.

The remaining centrifuged tubes containing the pellet and buffer were used for starch quantification. To extract the starch, the tubes were boiled at 100 °C for 10 min to allow starch gelatinization, and let sit for 20 min at room temperature (22 °C). Once cooled, 100 µL of amyglucosidase (7 units per mL, Sigma-Aldrich) and 100 µL amylase (0.7 units per mL, Sigma-Aldrich) were added to the tubes and incubated for 4 h at 37 °C in a rotating incubator. Samples were then centrifuged (10 min at 21,000*g*). Tubes were then centrifuged and 50 µL of supernatant was extracted and diluted in 1 mL of ultra-pure (UP) water (1:20, v:v) and vortexed. Total soluble sugar content was analyzed using the same method described above. Starch content was determined by subtracting the original from the post-digestion soluble sugar content. All samples were plated onto 96-well plates. To account for any procedural variability (chemicals, timing, pipetting, temperature, etc.), each plate contained a glucose standard curve (4 wells per plate) and wood/bark standard tissue samples with known soluble sugar and starch contents (4 wells of each per plate) that were concurrently undergoing all steps in the same chemical analysis. The wood/bark standard tissue is a sample from a homogenous mix of several thousand ground samples leftover from a 2016 preliminary part of the study^1^.

To calculate the coefficient of correlation (r) between yield and carbohydrate content for each of the 12 months preceding harvest (September till August) we used the ‘cor’ function using the Pearson method (R-core). A linear model Yield = ß_0_ + ß_1_ × Concentration_NSC type_ (lm, R-core) was used to estimate the slope parameter (ß_1;_ yield change in kg ha^−1^ in response to an increase of NSC concentration by 1 mg g^−1^ of tissue). To determine the most important months, for each carbohydrate type, in predicting the observed yields we used the Random Forest Regressor in PyCaret (Python; PyCaret.org. PyCaret, April 2020, URL https://pycaret.org/about. PyCaret version 2.3). The native function ‘feature importances’, in which ‘months’ were assigned as features, was used to indicate which months were the most important predictor of yield.

## Results

**Figure 2:**
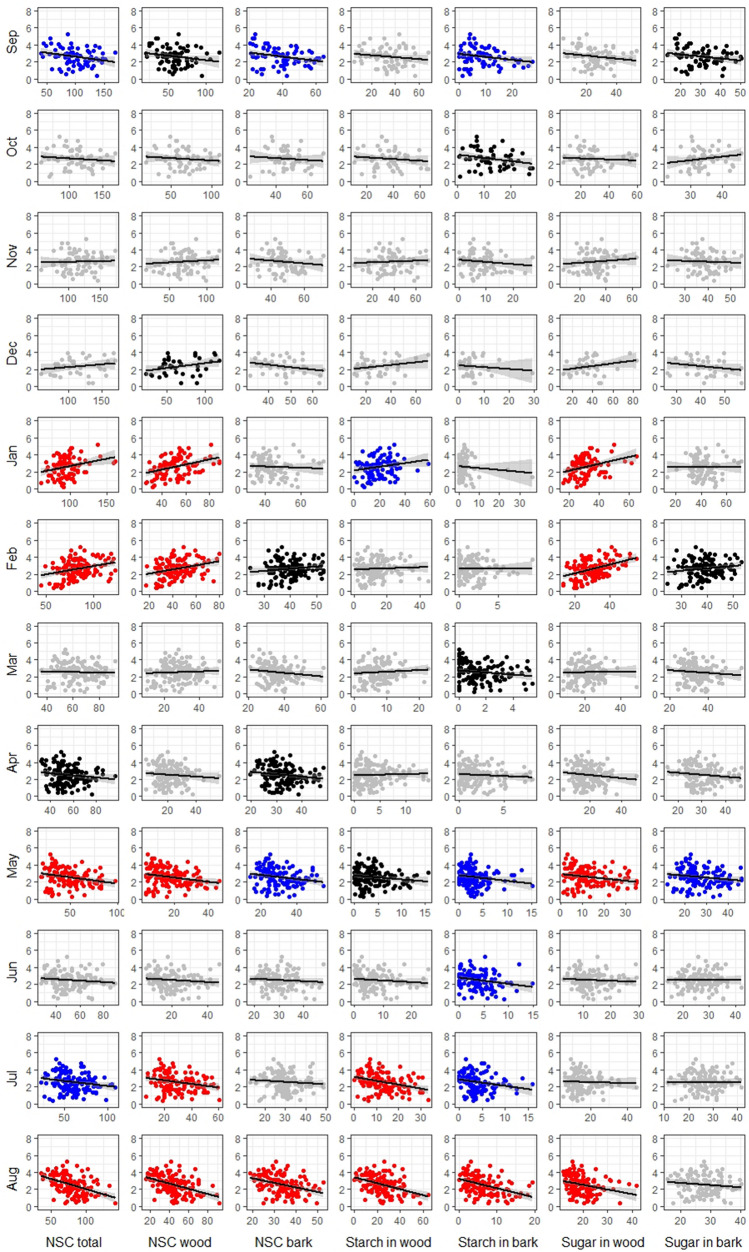
Correlations between yield (kg × 10^3^ ha^−1^) and carbohydrate concentration (mg g^−1^ of dry tissue) in twigs of *P. dulcis* during 12 months preceding the harvest. Level of correlation significance p-value reflected by graph color: red p-value < 0.01, blue p-value < 0.05, black p-value < 0.1, gray p-value > 0.1.

**Figure 3:**
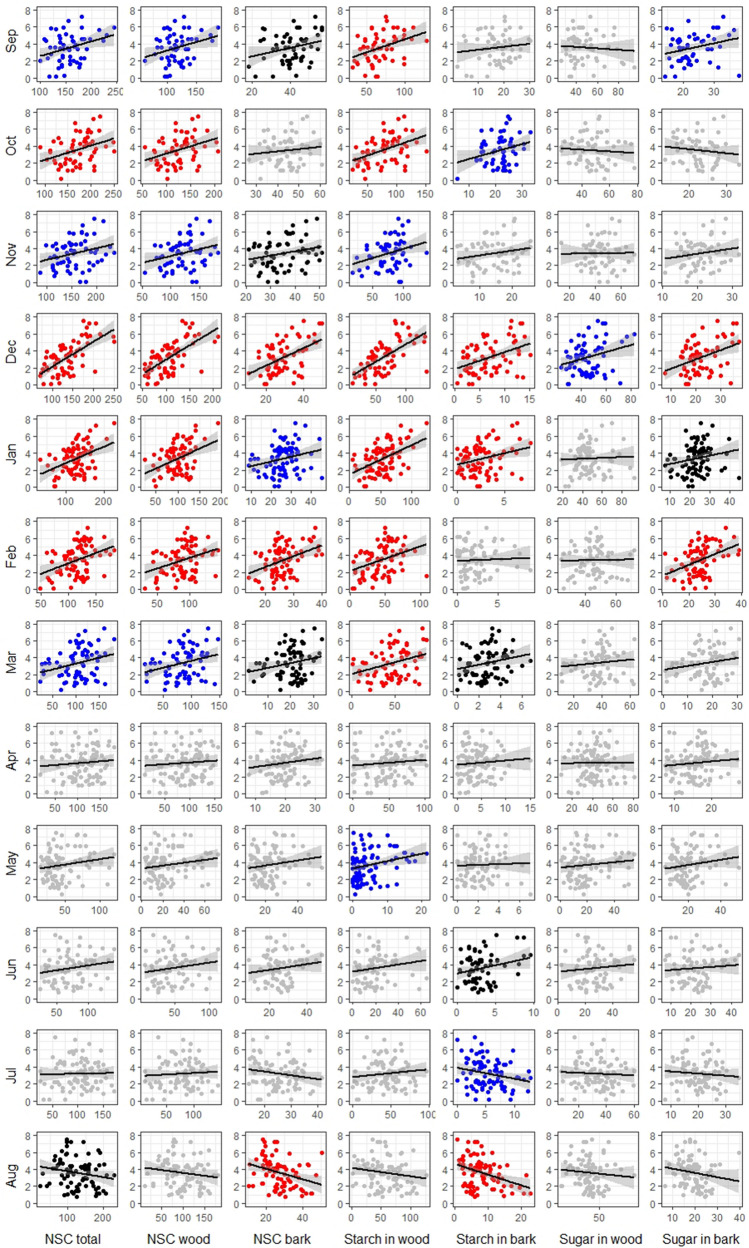
Correlations between yield (kg × 10^3^ ha^−1^) and carbohydrate concentration (mg g^−1^ of dry tissue) in twigs of *P. vera* during 12 months preceding the harvest. Level of correlation significance p-value reflected by graph color: red p-value < 0.01, blue p-value < 0.05, black p-value < 0.1, gray p-value > 0.1

**Figure 4:**
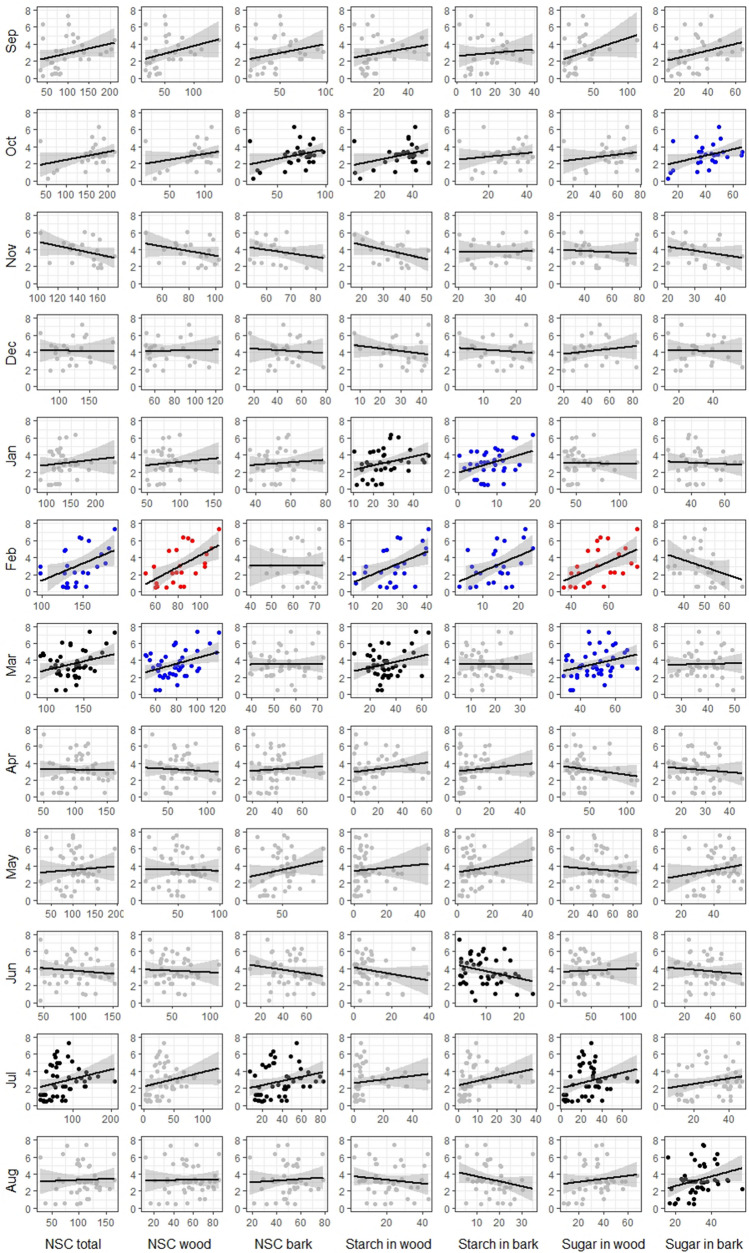
Correlations between yield (kg × 10^3^ ha^−1^) and carbohydrate concentration (mg g^−1^ of dry tissue) in twigs of *J. regia* during 12 months preceding the harvest. Level of correlation significance p-value reflected by graph color: red p-value < 0.01, blue p-value < 0.05, black p-value < 0.1, gray p-value > 0.1

From post-harvest (September) till harvest (August), NSC concentrations in twigs not only show seasonal variation^1^ but are also characterized by high variation within each month of the year in all three species (*P. dulcis*, *P. vera*, and *J. regia*; to access raw NSC data visit http://zlab-carb-observatory.herokuapp.com/). Due to the seasonal variation in NSC content, calculation of the coefficient of correlation between NSC content and yield was performed separately for each month. In general, the analysis revealed that the coefficients of correlation between NSC concentrations and yield were positive and significant (at p-value < 0.1) during mid-winter (January and February) in all three species (Table 1; Figs. 2, 3, 4). Moreover, *P. dulcis* (almond) was characterized by the presence of multiple periods of significant negative correlations between NSC content and yield; during the active period (April to July), highly significant negative correlations before harvest (August), and following harvest in September. Soluble sugar concentration in the bark was only weakly correlated with yield, while total NSC concentration and NSC concentration in wood was significantly correlated in 5 or 6 months during the year preceding harvest (Fig. 2). *P. vera* (pistachio) was characterized by significant positive correlations between concentrations of NSC and yield over the period from post-harvest till the end of dormancy (September till March). Specifically, starch content, in wood and bark, was the main driver behind these positive correlations. During the active period spanning from April till July, NSC contents were not significantly correlated with the current-year yield. A shift to negative correlations occurred in August (before harvest) when total NSC, NSC in bark, and starch in bark showed negative correlations with yield. Interestingly, starch content in wood and bark as well as the total content of NSC was most correlated with yield across all months, while soluble sugar concentration in wood remained uniformly non-correlated to yield through the entire season (Fig. 3). Out of the three analyzed species, NSC concentrations in *J. regia* (walnut) were the least correlated with yield. Only during the late dormancy period (February–March), the content of NSC and their forms were positively correlated with yield. Unlike in both other species, no significant negative correlations between NSC and yield were observed in *J. regia* (Fig. 4).

**Table 1:** Results from correlation analysis, *r* coefficient of correlation, *ß*_*1*_ slope of the correlation (yield change in kg ha^−1^ in response to an increase of NSC concentration by 1 mg g^−1^ of tissue), and *p-value* observed level of significance.

Month	NSC type	*P. dulcis*			*P. vera*			*J. regia*		
		r	ß_1_	p-value	r	ß_1_	p-value	r	ß_1_	p-value
Sep	NSC	− 0.257	− 9.13	2.53E−02	0.307	17.20	2.16E−02	0.281	11.26	1.19E−01
Sep	NSC in wood	− 0.201	− 10.61	8.14E−02	0.287	20.10	3.20E−02	0.288	18.06	1.11E−01
Sep	NSC in bark	− 0.285	− 23.99	1.25E−02	0.260	49.60	5.28E−02	0.230	21.78	2.05E−01
Sep	Starch in wood	− 0.152	− 11.31	1.89E−01	0.383	30.54	3.55E−03	0.197	28.90	2.80E−01
Sep	Starch in bark	− 0.232	− 34.74	4.37E−02	0.127	36.10	3.50E−01	0.094	19.81	6.10E−01
Sep	Sugar in wood	− 0.177	− 17.78	1.26E−01	− 0.078	− 8.57	5.68E−01	0.294	26.65	1.03E−01
Sep	Sugar in bark	− 0.211	− 24.17	6.76E−02	0.268	78.35	4.59E−02	0.281	39.83	1.19E−01
Oct	NSC	− 0.116	− 4.69	3.89E−01	0.361	16.80	5.36E−03	0.302	9.28	1.11E−01
Oct	NSC in wood	− 0.109	− 5.54	4.20E−01	0.352	17.33	6.79E−03	0.264	13.89	1.67E−01
Oct	NSC in bark	− 0.092	− 11.39	4.98E−01	0.116	26.47	3.77E−01	0.328	22.20	8.20E−02
Oct	Starch in wood	− 0.120	− 9.75	3.73E−01	0.431	23.98	7.38E−04	0.340	39.26	7.14E−02
Oct	Starch in bark	− 0.241	− 38.35	7.13E−02	0.267	87.87	3.90E−02	0.153	21.12	4.29E−01
Oct	Sugar in wood	− 0.061	− 5.70	6.50E−01	− 0.072	− 10.25	5.85E−01	0.178	15.40	3.55E−01
Oct	Sugar in bark	0.178	40.88	1.86E−01	− 0.120	− 47.17	3.62E−01	0.372	36.82	4.70E−02
Nov	NSC	0.037	1.77	7.53E−01	0.272	13.71	3.37E−02	− 0.340	− 24.93	1.43E−01
Nov	NSC in wood	0.090	4.83	4.48E−01	0.262	16.06	4.15E−02	− 0.294	− 27.10	2.08E−01
Nov	NSC in bark	− 0.122	− 16.57	3.05E−01	0.240	50.53	6.28E−02	− 0.245	− 42.07	2.97E−01
Nov	Starch in wood	0.058	4.61	6.25E−01	0.298	21.33	1.95E−02	− 0.359	− 56.61	1.20E−01
Nov	Starch in bark	− 0.128	− 25.87	2.80E−01	0.202	56.89	1.18E−01	0.004	0.85	9.85E−01
Nov	Sugar in wood	0.104	11.29	3.83E−01	0.015	2.34	9.09E−01	− 0.083	− 7.44	7.28E−01
Nov	Sugar in bark	− 0.048	− 9.11	6.85E−01	0.161	61.50	2.16E−01	− 0.257	− 45.26	2.75E−01
Dec	NSC	0.194	6.43	2.51E−01	0.636	29.46	1.22E−08	− 0.008	− 0.38	9.71E−01
Dec	NSC in wood	0.305	11.57	6.66E−02	0.626	33.93	2.48E−08	0.034	2.34	8.79E−01
Dec	NSC in bark	− 0.252	− 28.86	1.33E−01	0.481	103.95	4.44E−05	0.075	− 8.58	7.35E−01
Dec	Starch in wood	0.237	16.00	1.59E−01	0.626	40.35	2.45E−08	− 0.213	− 30.43	3.29E−01
Dec	Starch in bark	− 0.119	− 21.11	4.82E−01	0.425	198.60	3.71E−04	− 0.107	− 25.02	6.28E−01
Dec	Sugar in wood	0.266	15.62	1.11E−01	0.275	41.21	2.69E−02	0.171	14.74	4.37E−01
Dec	Sugar in bark	− 0.204	− 27.55	2.25E−01	0.380	110.40	1.63E−03	− 0.027	− 3.68	9.04E−01
Jan	NSC	0.272	17.21	6.56E−03	0.392	19.48	2.66E−04	0.118	6.65	4.93E−01
Jan	NSC in wood	0.374	29.70	1.39E−04	0.394	22.95	2.28E−04	0.111	7.88	5.14E−01
Jan	NSC in bark	− 0.057	− 7.65	5.78E−01	0.245	55.51	2.65E−02	0.092	14.93	5.92E−01
Jan	Starch in wood	0.203	21.22	4.43E−02	0.472	34.69	6.63E−06	0.307	52.02	6.45E−02
Jan	Starch in bark	0.093	− 22.08	3.61E−01	0.284	267.91	9.61E−03	0.357	130.61	3.25E−02
Jan	Sugar in wood	0.332	40.03	8.00E−04	0.040	4.80	7.17E−01	− 0.021	− 1.73	9.01E−01
Jan	Sugar in bark	0.001	− 0.11	9.93E−01	0.197	49.01	7.36E−02	− 0.058	− 8.49	7.34E−01
Feb	NSC	0.307	19.41	3.59E−04	0.375	24.55	7.08E−04	0.434	40.07	3.42E−02
Feb	NSC in wood	0.310	24.43	3.16E−04	0.335	24.95	2.70E−03	0.576	70.30	3.24E−03
Feb	NSC in bark	0.154	25.67	7.81E−02	0.397	127.60	3.18E−04	− 0.004	− 0.99	9.85E−01
Feb	Starch in wood	0.057	6.61	5.17E−01	0.358	29.12	1.29E−03	0.475	120.41	1.89E−02
Feb	Starch in bark	0.015	8.89	8.66E−01	0.042	39.22	7.16E−01	0.464	184.91	2.25E−02
Feb	Sugar in wood	0.422	51.76	5.27E−07	0.018	3.25	8.76E−01	0.516	93.80	9.84E−03
Feb	Sugar in bark	0.164	27.92	6.11E−02	0.396	128.52	3.33E−04	− 0.311	− 81.77	1.40E−01
Mar	NSC	− 0.031	− 2.69	7.16E−01	0.276	13.89	1.82E−02	0.288	20.28	5.27E−02
Mar	NSC in wood	0.056	6.79	5.11E−01	0.274	16.18	1.90E−02	0.368	33.98	1.19E−02
Mar	NSC in bark	− 0.130	− 20.47	1.27E−01	0.218	56.67	6.45E−02	0.016	2.82	9.14E−01
Mar	Starch in wood	0.085	19.41	3.23E−01	0.329	27.07	4.52E−03	0.260	33.80	8.09E−02
Mar	Starch in bark	− 0.160	− 119.89	6.01E−02	0.217	267.36	6.58E−02	− 0.005	− 1.26	9.73E−01
Mar	Sugar in wood	0.015	2.40	8.59E−01	0.105	16.88	3.79E−01	0.302	45.99	4.15E−02
Mar	Sugar in bark	− 0.095	− 15.82	2.68E−01	0.175	46.74	1.39E−01	0.029	7.01	8.51E−01
Apr	NSC	− 0.142	− 12.47	8.02E−02	0.093	4.32	3.55E−01	− 0.017	− 0.99	9.07E−01
Apr	NSC in wood	− 0.089	− 11.27	2.76E−01	0.083	4.06	4.12E−01	− 0.061	− 4.96	6.65E−01
Apr	NSC in bark	− 0.159	− 27.21	5.09E−02	0.130	49.19	1.95E−01	0.066	9.03	6.42E−01
Apr	Starch in wood	0.045	15.23	5.85E−01	0.104	6.73	3.01E−01	0.179	18.38	2.05E−01
Apr	Starch in bark	− 0.080	− 46.50	3.29E−01	0.077	50.18	4.40E−01	0.125	18.43	3.78E−01
Apr	Sugar in wood	− 0.123	− 17.74	1.30E−01	0.010	1.36	9.19E−01	− 0.173	− 11.97	2.19E−01
Apr	Sugar in bark	− 0.132	− 22.70	1.04E−01	0.095	41.22	3.43E−01	− 0.090	− 23.29	5.28E−01
May	NSC	− 0.253	− 14.89	3.13E−03	0.175	12.91	1.06E−01	0.069	4.25	6.40E−01
May	NSC in wood	− 0.247	− 24.69	3.82E−03	0.162	16.82	1.34E−01	− 0.025	− 2.41	8.68E−01
May	NSC in bark	− 0.208	− 24.01	1.54E−02	0.155	29.64	1.53E−01	0.180	23.43	2.20E−01
May	Starch in wood	− 0.156	− 45.43	7.15E−02	0.242	86.27	2.37E−02	0.085	18.64	5.65E−01
May	Starch in bark	− 0.175	− 66.32	4.21E−02	0.037	44.37	7.35E−01	0.125	34.29	3.97E−01
May	Sugar in wood	− 0.226	− 26.40	8.36E−03	0.117	15.84	2.79E−01	− 0.080	− 9.65	5.87E−01
May	Sugar in bark	− 0.172	− 22.00	4.55E−02	0.153	30.53	1.57E−01	0.176	33.15	2.33E−01
Jun	NSC	− 0.119	− 8.36	1.99E−01	0.181	11.50	1.34E−01	− 0.120	− 6.58	4.38E−01
Jun	NSC in wood	− 0.114	− 12.19	2.16E−01	0.164	12.44	1.74E−01	− 0.046	− 3.71	7.65E−01
Jun	NSC in bark	− 0.094	− 14.47	3.09E−01	0.148	32.75	2.21E−01	− 0.179	− 20.01	2.44E−01
Jun	Starch in wood	− 0.114	− 19.63	2.16E−01	0.176	20.65	1.45E−01	− 0.200	− 37.57	1.92E−01
Jun	Starch in bark	− 0.218	− 74.61	1.74E−02	0.228	195.45	5.75E−02	− 0.286	− 86.73	5.97E−02
Jun	Sugar in wood	− 0.072	− 13.12	4.34E−01	0.103	15.88	3.98E−01	0.044	3.88	7.79E−01
Jun	Sugar in bark	0.003	0.53	9.75E−01	0.084	17.95	4.89E−01	− 0.094	− 13.17	5.46E−01
Jul	NSC	− 0.207	− 11.92	1.64E−02	0.025	1.21	8.21E−01	0.257	11.04	7.83E−02
Jul	NSC in wood	− 0.248	− 20.96	3.83E−03	0.065	3.61	5.50E−01	0.235	17.19	1.08E−01
Jul	NSC in bark	− 0.096	− 14.06	2.69E−01	− 0.172	− 43.93	1.12E−01	0.260	24.51	7.43E−02
Jul	Starch in wood	− 0.348	− 45.24	3.71E−05	0.120	9.22	2.70E−01	0.138	21.01	3.51E−01
Jul	Starch in bark	− 0.220	− 75.68	1.07E−02	− 0.225	− 129.61	3.61E−02	0.240	49.09	1.00E−01
Jul	Sugar in wood	− 0.038	− 5.65	6.62E−01	− 0.052	− 7.13	6.32E−01	0.262	30.02	7.20E−02
Jul	Sugar in bark	− 0.001	− 0.22	9.89E−01	− 0.082	− 25.13	4.49E−01	0.226	32.12	1.23E−01
Aug	NSC	− 0.458	− 24.43	2.27E−08	− 0.184	− 6.98	9.59E−02	0.041	2.49	7.90E−01
Aug	NSC in wood	− 0.439	− 30.81	1.03E−07	− 0.151	− 6.79	1.74E−01	0.018	1.74	9.05E−01
Aug	NSC in bark	− 0.355	− 53.48	2.41E−05	− 0.311	− 61.24	4.02E−03	0.068	9.55	6.59E−01
Aug	Starch in wood	− 0.412	− 35.28	6.76E−07	− 0.159	− 9.81	1.52E−01	− 0.133	− 22.24	3.82E−01
Aug	Starch in bark	− 0.434	− 109.77	1.41E−07	− 0.360	− 124.83	7.69E−04	− 0.227	− 58.67	1.34E−01
Aug	Sugar in wood	− 0.281	− 55.29	9.64E−04	− 0.093	− 11.23	4.02E−01	0.134	18.16	3.79E−01
Aug	Sugar in bark	− 0.128	− 25.86	1.38E−01	− 0.164	− 49.96	1.36E−01	0.276	56.20	6.68E−02

**Figure 5:**
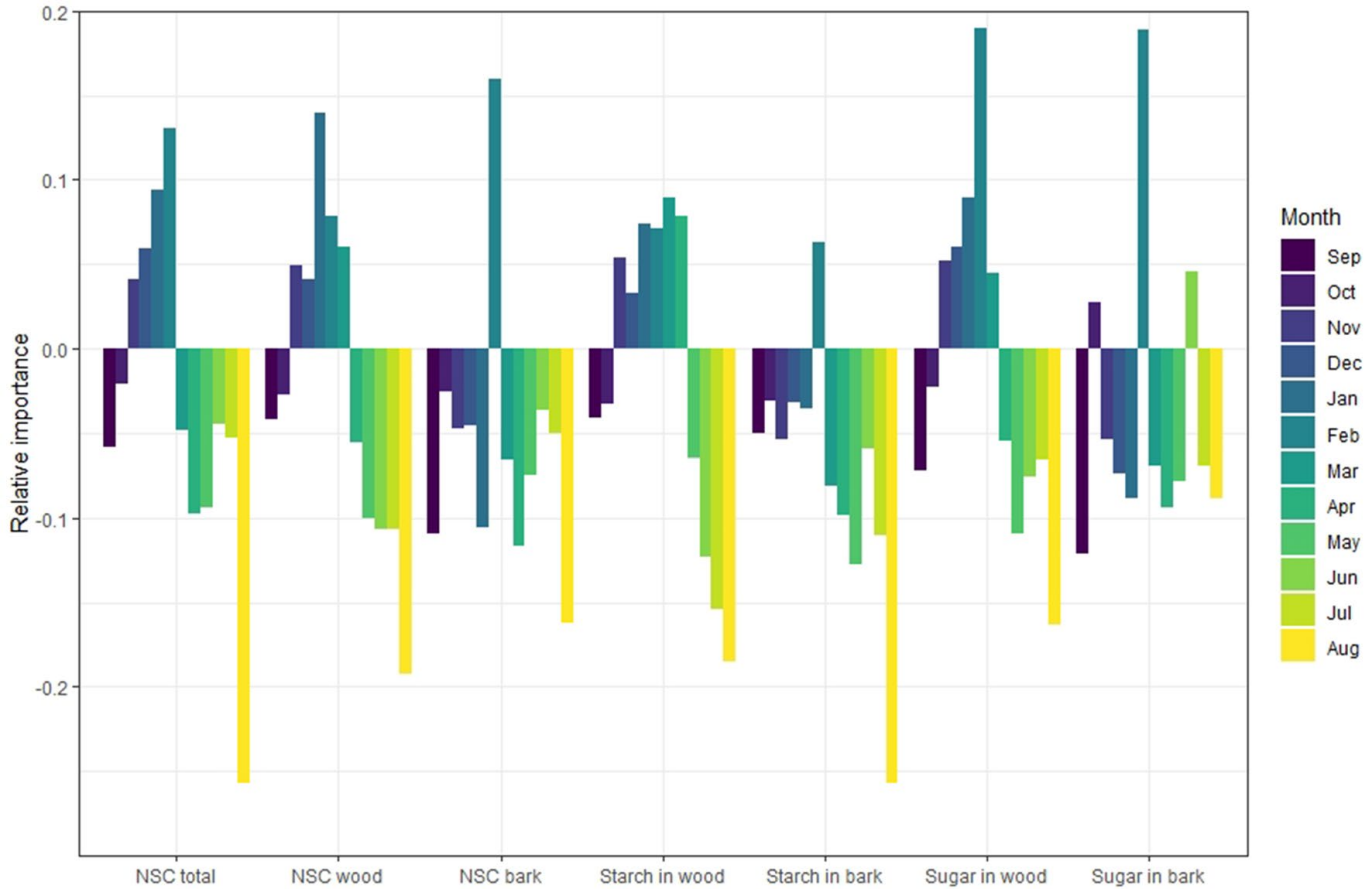
Relative importance of each month’s carbohydrate concentration on realized yield in *P. dulcis*. Positive importance reflects positive correlation and negative importance reflects negative correlation between NSC and yield.

**Figure 6:**
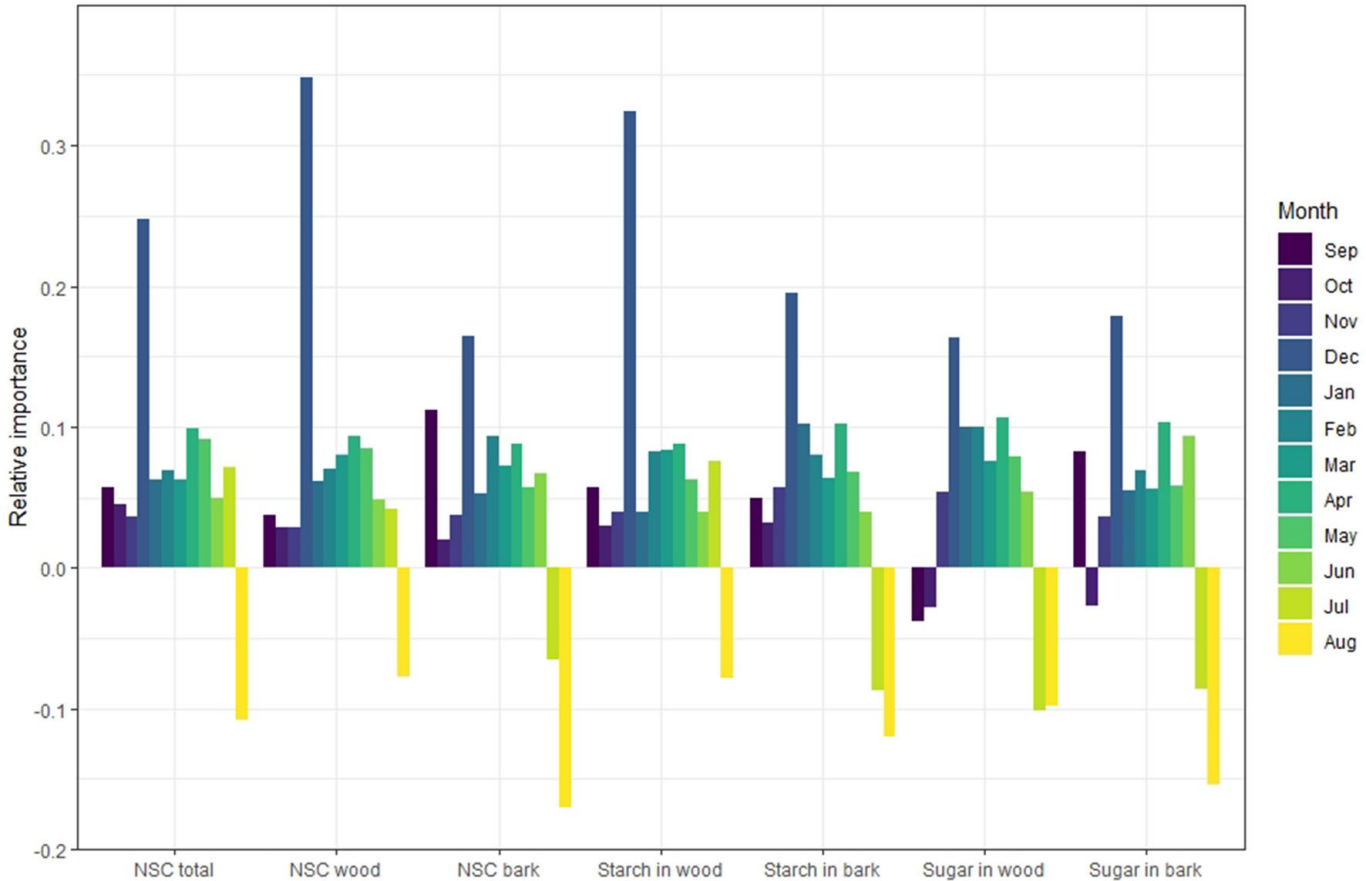
Relative importance each month carbohydrate concentration on realized yield in *P. vera*. Positive importance reflects positive correlation and negative importance reflects negative correlation between NSC and yield.

**Figure 7:**
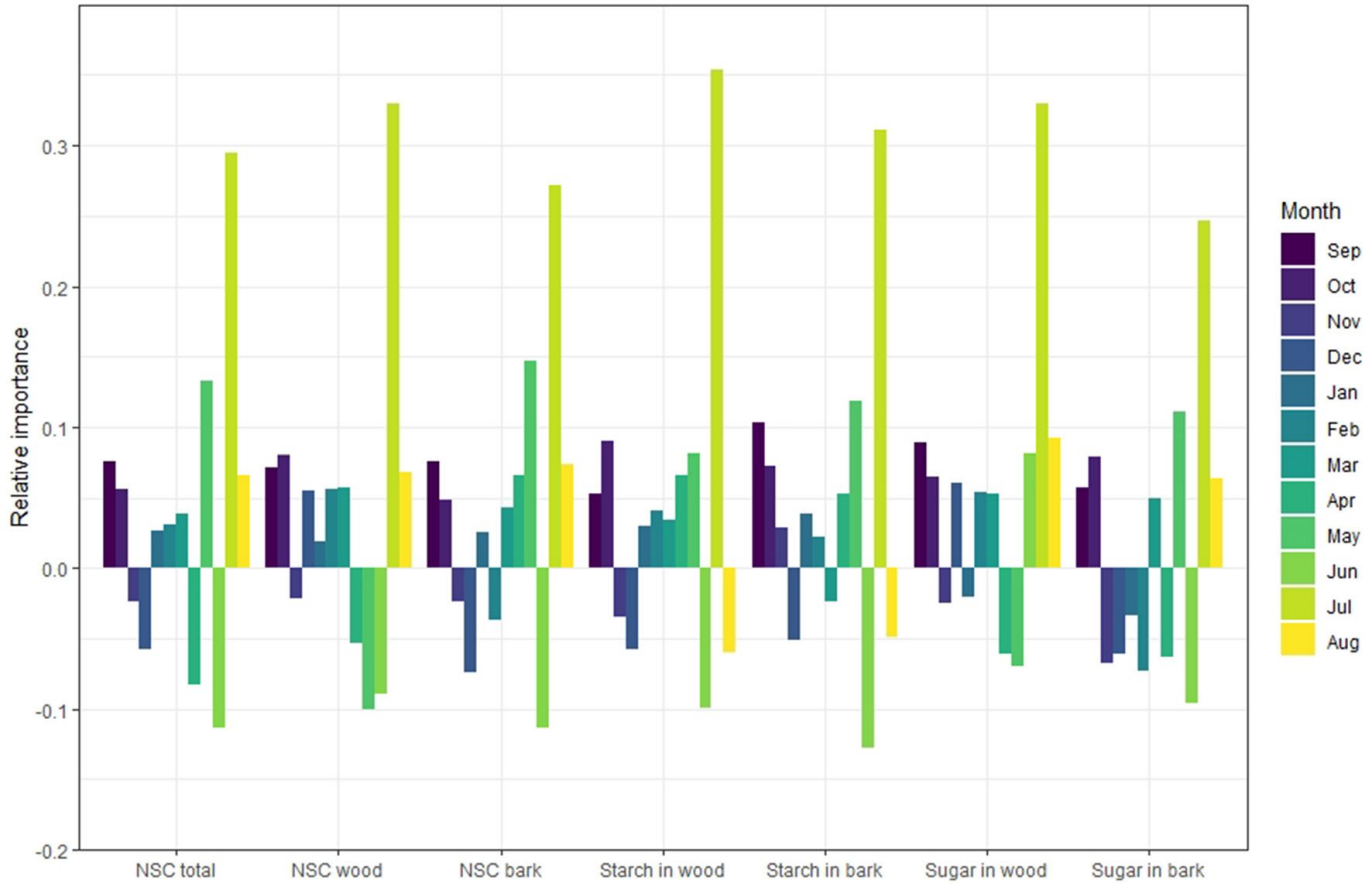
Relative importance each month carbohydrate concentration on realized yield in *J. regia*. Positive importance reflects positive correlation and negative importance reflects negative correlation between NSC and yield.

Each type of carbohydrate (NSC total, NSC in wood, NSC in bark, starch in wood, starch in bark, soluble sugars in wood, and soluble sugars in bark) was independently analyzed and the relative importance of each month’s content as the predictor of yield was determined using the Random Forest Regressor algorithm (PyCaret’s Regression Module with the split between training and test group of 0.7 and 0.3 respectively; Figs. 5, 6, 7). The positive or the negative impact of NSC concentrations on yield was assigned using the sign of coefficient of correlation for that month (Table 1). In *P. dulcis*, NSC content in February was the most important positive feature contributing to yield, while NSC concentration in August was the most important negative feature in predicting yield (Fig. 5). In *P. vera*, NSC concentrations in December was the most important positive feature in predicting yield, while NSC concentrations in August was the most important negative indicators predicting high yield (Fig. 6). In *J. regia*, NSC concentration in July was the most important positive indicator for yield followed by May concentrations, while the concentration in June was the most important negative feature in predicting yield (Fig. 7).

## Discussion

The two main goals of the presented work were (1) to test for the presence of correlations between NSC concentration in twigs of nut trees and orchards’ yields and (2) to determine the months at which carbohydrate concentration were the best predictors of realized yield. In general, several correlations were significant (Table 1), the coefficient of correlation (r) ranged from maximum positive correlations of 0.42, 0.63, and 0.52 and negative correlations of − 0.44, − 0.36, and − 0.36 for *P. dulcis*, *P. vera*, and *J. regia*, respectively. Typically, winter (dormancy) NSC concentration was positively correlated with yield, while the summer (active period) NSC concentration was negatively correlated with yield. Thus, more is not always better. In fact, the apparent exhaustion of NSC just prior to harvest can be linked to high yield, in which case, less is better. The presence of such correlations underlines the importance of monitoring NSC reserves for enhancing yield success.

The inverse relationship between yield and summer NSC suggests that yield comes at the expense of NSC reserve formation. Nevertheless, the positive correlation in the fall and winter requires that NSC reserves be replenished during the short postharvest period, prior to senescence, to assure adequate reserves for bloom. If NSC reserves are not replenished, a lower yield may be expected and may help explain the presence of alternate bearing at either the whole-tree or orchard level in the case of *P. vera*^16^ or at the twig level seen in *P. dulcis*^17^. Interestingly, despite the general trends mentioned above, there were large differences in the magnitude and temporal patterns of the positive and negative impacts of high NSC levels on yield among the studied species. In *P. vera,* NSC levels were almost always positively correlated with yield from September through June (i.e. through the post-harvest, dormancy period, bloom, and vegetative part of the season). Out of all the months however, December NSC concentration was the most important positive predictor of yield, this was true in all of its studied forms and locations (NSC total, NSC in wood, NSC in bark, starch in wood, starch in bark, soluble sugars in wood and soluble sugars in bark). NSC concentration was only negatively correlated with yield in the short period preceding fruit maturation (August). The low levels of August NSC associated with high yields, suggest that reserve exhaustion during this period was correlated with a high accumulation of nut biomass. This pattern may reflect sink dominance of fruit over reserve formation. However, if high yield results in the depletion of NSC concentrations to the extent that they cannot be replenished prior to senescence (high levels of NSC are required in December to assure high yield), then this may lead to a reduction in the following year’s yield and ultimately explain the alternate bearing habit seen in *P. vera*^16^. If true, breeding objectives aiming to reduce alternate bearing in *P. vera*^18^ may benefit from selecting varieties that show a strong NSC recovery pattern in the fall.

*Prunus dulcis* presents a slightly more complicated picture of carbohydrates’ impact on yield. NSC total, starch, and sugar concentrations in wood were positively correlated with yield in late fall and during dormancy (November through February) with February reserves being the most important positive feature associated with high yield. This would suggest that high NSC levels just prior to and during bloom are the most important prerequisite for achieving higher yields. Hence, a high NSC content during dormancy, achieved either by preservation and/or by the influx of sugars from more distal sources during bloom, to provide sufficient energy and structural material for flowers is the key element to yield success. This finding may also provide an interesting opportunity for *P. dulcis* breeding efforts, wherein selection could be informed by high NSC levels in February^19^. The sudden change in direction, from a positive relationship in February to a negative in March, most likely reflects the strong dependence on local twig reserves for sustaining a healthy bloom and promoting vegetative bud pushing. The ensuing negative correlation, characterized by a steep decline in NSC concentration beginning in March, continuing through the summer^1,3^ suggests that during the most active period, the reproductive NSC sink takes precedence over reserve formation. While reproductive prioritization outweighs reserve formation in both *P. dulcis* and *P. vera,* the persistent decline observed in *P. dulcis* comes in stark contrast to *P. vera,* where only a narrow time frame, the nut filling period, was negatively correlated with yield. This prolonged period of low NSC content and its associated negative correlation with yield in *P. dulcis* may be offset by the fact that while *P. dulcis* has the earliest harvest amongst the three species, its senescence occurs at the same time as in *P. vera* and *J. regia.* Thus, in effect allowing more time for the recovery of NSC and potentially avoiding a pronounced alternate bearing habit^17^.

*Juglans regia* presents the most ambiguous pattern of carbohydrate impact on yield. Much like *P. dulcis* and *P. vera*, we also found the strongest positive correlations between NSC content and yield in the months just prior to budbreak. However, in contrast to the two-former species, *J. regia* is a wind-pollinated species with female flowers developing on new vegetative extension growth and thus relies on the concurrent development of both the vegetative and flowering structures. As a result, this magnifies the burden of bearing enough NSC reserves to initiate both their growths following dormancy. In walnut, this burden exceeds the storage-supply capacity within the twigs and it must therefore import NSC from distant sources to attain sufficient energy. Therefore, in preparation for bloom, *J. regia* may strongly depend on the redistribution of NSC, via the xylem, from the stem to twigs thus reducing its reliance on autumnal carbohydrate reserves. Indeed, in February and March, we observed that increases in twig NSC were most consistently in the woody conductive tissues^15,20^. Furthermore, given that flowering and vegetative growth occur simultaneously, photosynthetic independence promptly follows thereby quickly becoming the main energy source for supporting both their growths. Hence, the dependence on a distal energetic supply for growth initiation and then on current photosynthate for growth sustenance may explain the lack of significant correlations between twig NSC storage and yield. As a consequence, the antagonistic relationship between storage and reproductive sinks that is more apparent in *P. vera* and *P. dulcis* is diminished, making *J. regia* potentially less sensitive to twig reserve carbohydrate content. Interestingly, when analyzing monthly importance there were consecutive shifts; from a high NSC content in June, as the most important predictor of low crop, to a high NSC content in July, as the most important predictor of high yield. These months, in particular, coincide with the transitional phases that occur between growth and storage accumulation. In *J. regia* specifically, June marks the fastest growth rates and the lowest carbohydrates content while July is the moment at which growth slows but also the point of maximum reserve accumulation rates^1^. Therefore, in the context of phenology and yield, it is thought-provoking that the crux between the interplay of these months is captured as most important for predicting final yields. Such increasing importance on mid-summer carbohydrate concentrations further supports the notion that in *J. regia*, high crop is not dependent on the competition between storage and yield but rather on an overall high photosynthetic productivity especially at the end of summer. The low dependence of yield on autumnal NSC reserves can be expected from the fact that multi-year observations of NSC content on walnut tree twigs were relatively unaffected by seasonality compared to the two other studied species^1^. In addition, it is important to note that an impact of NSC reserves on yield is not always detected, for example in *Olea europea* L. (olive tree) no such impact has been reported^21^. However, such a relationship may be very difficult to detect in small, short term experimental studies due to high temporal and year-to-year variation in NSC content^1,3,9^.

In all cases, only 1 or 2 months shared a high importance for crop prediction and such distribution of importance may suggest the practical implications in using carbohydrate analyses for orchard management and decision making. A simple NSC concentration analysis in twigs at specific months for example, December for *P. vera* or February for *P. dulcis*, can help project yield and provide information for assessing irrigation and fertilization needs. As non-structural carbohydrate content represents a buffer between photosynthetic capacity and needs (base respiration, growth, yield, defense, and dormancy reserve formation), knowledge on their dynamics may provide physiological insights to better understand the physiological status of trees. Sudden and unexpected changes to NSC concentrations may reflect orchard health issues. The introduction of NSC analysis to breeding may open new avenues in the search for high-yielding varieties. We can also expect that adding NSC content analysis to yield prediction models which consider environmental elements (temperature, rainfall) as physiological attributes and encompass a range of abiotic and biotic stressors (tree water status, pathogen infestations, fertilization, etc.) will improve their performance.
